# Pharmacological Intervention of Nicotine Dependence

**DOI:** 10.1155/2013/278392

**Published:** 2013-12-29

**Authors:** Raka Jain, Pradipta Majumder, Tina Gupta

**Affiliations:** ^1^National Drug Dependence Treatment Centre, All India Institute of Medical Sciences, Ansari Nagar, New Delhi 110029, India; ^2^Department of Psychiatry, National Drug Dependence Treatment Center, All India Institute of Medical Sciences, New Delhi 110029, India

## Abstract

Nicotine dependence is a major cause of mortality and morbidity all over the world. Various medications have been tried to treat nicotine dependence including nicotine replacement therapy, bupropion, and varenicline. A newer venture to nicotine dependence treatment is a nicotine vaccine which is yet to get footsteps in common practice. The present review assimilates various pharmacotherapeutic measures to address nicotine dependence. However, it is to be noted that psychological interventions, when combined with pharmacotherapy, offer the greatest benefits to the patients.

## 1. Introduction

Worldwide there are nearly 1.2 billion users of nicotine and tobacco products [1]. In India, the overall prevalence of current tobacco use from the NHSDAA (National Household Survey of Drug and Alcohol Abuse) was 55.8% [2]. Worldwide tobacco use causes more than 5 million deaths per year, and if smoking prevalence continues to increase in the developing world, the number of annual deaths attributable to cigarette smoking will be more than 8 million by 2030 [1]. Eleven percent of deaths from ischemic heart disease, the world's leading killer, are attributable to tobacco use. More than 70% of deaths from lung, trachea, and bronchus cancers are attributable to tobacco use [3].

Across studies, it has been found that the morbidity and mortality associated with tobacco use are substantially reduced by complete cessation of smoking [4]. It is important for all clinicians to make a rigorous effort to motivate tobacco users to cease tobacco use and to assist in their effort to quit [4]. The Clinical Practice Guideline on Treating Tobacco Use and Dependence published by the US Public Health Service recommends to *ask* the patient if he or she uses tobacco, *advise* him or her to quit, *assess* willingness to make a quit attempt, *assist* him or her in making the quit attempt, and *arrange* for follow-up contacts to prevent relapse [4]. In a meta-analysis, it was shown that brief advice to quit smoking from a clinician increases cessation rates by 30% [4].

The risk reduction after quitting smoking varies according to the disease under consideration and also the population concerned. It is found that risk of cardiovascular disease-related death decreases precipitously at 6 months to 2 years. In case of lung diseases and various cancers, the reduction is less pronounced and more gradual. Improvements in lung function can be discerned as soon as 1 year after cessation, and with sustained abstinence, the age-related decline in lung function returns to that of nonsmokers [5]. In case of pregnant women, the risks of smoking-related pregnancy complications are reduced to almost the nonsmoker level if they quit during the first trimester [5]. The significant risk reduction for cancers after stopping smoking can be seen in 5 to 15 years, though the risk usually does not appear to reach the level of never smokers [5].

## 2. Why Pharmacotherapy for the Treatment of Nicotine Dependence?

Pharmacotherapy has been of proven benefit in treating nicotine dependence. It is also recommended that pharmacotherapy should be offered to “all smokers trying to quit, except in the presence of special circumstances.” [4]. However, with selected populations: those with medical contraindications, those smoking fewer than 10 cigarettes per day, pregnant/breastfeeding women, and adolescent smokers, special consideration should be given before using pharmacotherapy [4]. The most commonly used pharmacotherapy in case of nicotine dependence is the nicotine replacement therapy (NRT). The current clinical practice guideline recommends that NRT should be used by all smokers who are trying to stop smoking [6]. NRT is generally considered safe intervention to general populations and higher-risk groups, including pregnant and breastfeeding women, adolescents, and smokers with cardiovascular disease [7]. In a meta-analysis it was found that compared with placebo twice the number of smokers sustained six months' abstinence as a result of nicotine replacement therapy [8]. The first-line pharmacotherapies include nicotine replacement medications, bupropion and varenicline which are all US FDA approved. Clonidine and nortriptyline are suggested as the second line agents [4].

However, current pharmacological therapies available to curb nicotine addiction offer only limited success [9]. One reason for the low success is that many quitting attempts are unplanned so that the most effective cessation aids may not be used [8].

The main conclusions from the recently updated US guidelines [4] for the treatment of tobacco dependence are as follows.

1. The role of counseling as a modality of treatment in nicotine dependent individuals is more important than that thought previously.
2. Varenicline and nicotine patches in combination with an oral product are possibly the most effective pharmacological treatments.
3. All smokers, irrespective of their intention to quit, should be provided with the benefit of brief interventions.
4. There is a dearth of evidence to endorse the use of medications by adolescents, pregnant smokers, light smokers (<10 cigarettes per day), or smokeless tobacco users.

## 3. Nicotine Replacement Therapy (NRT)

Nicotine, possessing an alkaloid structure, is mainly present in the leaves of *Solanaceae *plants such as tobacco [9]. Nicotine is produces dependence by activating mesolimbic dopaminergic reward system. Nicotine acts as an agonist of neural nicotinic acetylcholine receptors (NAChRs), which are found presynaptically in the central nervous system and postsynaptically in the autonomic nervous system [6]. With the increase in exposure to nicotine, NAChRs also are increased, which results in nicotine tolerance. Thus, those factors that decrease bioavailability of nicotine are hypothesized to increase cravings for tobacco and decrease the likelihood of cessation because more of the drug is needed to achieve a given level of dopamine. Extrapolating this has led to the development of smoking cessation treatment methods that emphasize nicotine replacement [6, 10].

The rationale of using nicotine replacement medication for treating nicotine dependence is based on the theory of harm reduction. The aim of the therapy is to relieve the withdrawal symptoms related to nicotine use and thus help the client to quit. Nicotine-containing medications make it easier to abstain from tobacco by replacing the nicotine formerly obtained from tobacco and thereby providing nicotine-mediated neuropharmacologic effects [11]. The nicotine-replacement medications reduce the withdrawal symptoms or at least prominent ones, thus helping people to function without cigarettes. The medications may also reduce the reinforcing effects of tobacco-delivered nicotine. Finally, nicotine medications may provide at least some effects for which the patient previously relied on cigarettes, like sustaining desirable mood and attention states, making it easier to handle stressful or boring situations. However the evidence for the operation of these mechanisms is not conclusive. Nonetheless, all of the approved nicotine replacement medications have been found to be safe and effective in smoking cessation [11].

The most common indication for NRT is to aid the patients in abrupt cessation, but additional uses are also licensed in some countries, such as gradual reduction to quit; temporary abstinence, that is, for short periods of abstinence where smoking is not allowed; and maintenance of reduction [12]. NRT has also been investigated as an aid in reduction of habit size of nicotine use among smokers not willing to quit. In 2 meta-analyses, it was found that for smokers who are unwilling to stop smoking completely but want to reduce their habit size the use of NR compared with placebo increased the likelihood that they would make a cessation attempt and that they succeeded more often with the cessation [4, 12].

NRTs are well tolerated and no life threatening adverse events have been noted in studies. In a recent review, nausea was found to be more common than placebo [8]. In another review, it was found that NRT is associated with an increased risk of gastrointestinal complaints and insomnia. There were also increased risk of skin irritation with the nicotine patch and oropharyngeal complaints with orally administered NRT [7]. The most serious adverse events, consistently reported in both RCTs and observational studies, were heart palpitations, chest pains, and other arrhythmias including atrial fibrillation and myocardial infarction [7]. Unofficial guidelines recommend cautious use of NRT in patients with known cardiovascular disease in the absence of a physician [13].

## 4. Currently Available Nicotine Replacement Therapies

Nicotine replacement medications should not be viewed as standalone medications that make people stop smoking. Reassurance and guidance from health professionals can be critical to achieve and sustain abstinence. Six types of nicotine replacement products are on the market. These include nicotine transdermal patch systems; nicotine nasal spray; and nicotine delivery through the oral mucosa including gum, lozenge, sublingual tablet, and vapor inhaler [11]. Two-milligram nicotine gum was first introduced in the United States in February 1984 and was available to the consumers only with prescription [6]. Prescription-only nicotine patches were introduced in 1992, followed by a nasal spray (1996), inhaler (1997), and lozenge (2003) [6]. Common adverse events seen with all NRT products include dizziness, nausea, and headache.

## 5. Transdermal Nicotine Patches

Nicotine patches deliver nicotine through the skin at a relatively steady rate [14]. Currently, four different types of patches are available in the market with variation in their design, pharmacokinetics, and duration of wear (i.e., 24- and 16-hour wear). The dose adjustment may be done depending upon the habit size of the patients. For example, the NicoDerm CQ patch (marketed in the United States by GlaxoSmithKline Consumer HealthCare) has 7, 14, and 21 mg/day dose strengths and has been shown effective in both 16- and 24-hour uses. Smokers who use 10 or less cigarettes per day are instructed to begin with the 14 mg patch, and those who smoke more than 10 per day are instructed to start with 21 mg [11]. Moreover, the NicoDerm CQ and Habitrol systems are designed to be worn for 24 hours, but they can be removed after 16 hours and the Nicotrol system is designed for 16 hours of wear (subjects are instructed to remove the patch at bedtime). Use of the patch overnight may have advantage in relieving morning craving but may be more likely to induce sleep disturbances. In a clinical trial which compared the NicoDerm CQ patch (21 mg/24 hours) to the Nicotrol patch (15 mg/16 hours), it was found that the 21 mg/24-hour patch yielded consistently better control of craving, not only during the morning hours but also throughout the day and over the 2-week period of abstinence [15]. However, increasing nicotine uptake by using higher doses of transdermally absorbed nicotine has yielded marginally higher success rates [16, 17].

The advantage of nicotine transdermal patches over other preparations could be the ease to ensure compliance to medication [2]. However, the patches may not be adequate in relieving “acute” craving provoked by smoking-related stimuli for all smokers [11]. A study tested the efficacy of nicotine patches in combination with behavioral therapy for the treatment of adolescent spit tobacco addiction. Tobacco cessation rates were examined in three treatment groups: a usual care group, a behavior intervention with placebo patch group, and a behavior intervention with active nicotine patches group. The tobacco cessation rate for the usual care group was 11.4%; for the placebo patch group, 25.0%; and for active patch, 17.3%. The cessation rates for active and placebo patches were not significantly different, proving that behavioral intervention is twice as successful and that nicotine patch did not offer additional improvement [18]. For breakthrough cravings not adequately controlled by transdermal nicotine alone, acute therapies may be added.

## 6. Nicotine Gum

Nicotine gum, the first NRT, was first made available in Europe in the early 1980s and in the US in 1984 [11]. The gum is available in different flavors. The gum is available in two doses: 2 mg and 4 mg, delivering approximately 1 mg and 2 mg, respectively. Users are instructed to use a piece of gum every 1-2 hours for the first 6 weeks, then to reduce use to one piece every 2–4 hours for 3 weeks, and one piece every 4–8 hours for 3 weeks. In highly dependent smokers, the 4 mg gum is superior to the 2 mg gum. Since about 50% of the nicotine in gum is absorbed, a fixed schedule of 10 pieces per day, a smoker receives about 10 mg or 20 mg of nicotine per day using the 2 mg or 4 mg gum, respectively. Round about 50% of the nicotine is absorbed through buccal mucosa [19]. Moreover because of the slow absorption through buccal mucosa the highest arterial level of nicotine attained after using gum is relatively less than attained by smoking cigarette. It has also been seen that using acidic beverages prior to the use of the gum interferes with its absorption; patients should avoid acidic beverages (e.g., soda, coffee, and beer) for 15 minutes before and during chewing gum [11]. Nicotine gum chewing may cause jaw soreness; therefore, the smoker should chew the gum to release nicotine, and then move the gum between the cheek and gum for a minute or so. Gum can also cause a mild burning sensation in the mouth and throat, which may be undesirable.

## 7. Lozenge

Nicotine lozenge is available in the market in 1 mg, 2 mg, and 4 mg strengths [20]. The lozenge, unlike the gum, should not be chewed and is considered both a benefit by some patients and a weakness by others who enjoy gum chewing. The amount of nicotine delivered per lozenge is higher than that delivered by gum. Single dose studies demonstrated 8% to 10% higher maximal plasma concentration and 25% to 27% higher AUC values (area under concentration-time curve) from lozenges compared with gums at both 2 and 4 mg dose levels [21].

## 8. Inhaler

*This is a prescription medication in USA unlike gum*. The inhaler device consists of a mouthpiece and a plastic cartridge which contains nicotine. Puffing the inhaler draws nicotine through the mouthpiece into the mouth of the smoker. This design particularly aimed at satisfying the hand-to-mouth ritual of smoking [11]. The amount of nicotine delivered through this device is related to the number of inhalations. 80 deep puffs of the inhaler deliver 4 mg of nicotine. However, depth of inhalation is not a major determinant of dosing [22]. Rather, the amount of nicotine through the inhaler is temperature-dependent, with higher ambient air temperatures delivering larger amounts of nicotine and lower temperatures delivering smaller amounts [23]. The product is not a true inhaler; nicotine is not delivered to the bronchi or lungs, but rather deposited and absorbed in the mouth, much like nicotine gum [11]. Most people use between 6 and 16 cartridges a day, and the recommended duration of treatment is 3 months, after which patients may be weaned by gradual reduction over the following 6–12 weeks.

## 9. Nasal Spray

Marketed as a prescription medication, the nasal spray is designed to deliver nicotine more rapidly than other NRTs and provides acute craving relief. Nicotine nasal spray is an aqueous solution of nicotine in a 10 mL spray bottle [23]. It consists of a multidose bottle with a pump that delivers 0.5 mg of nicotine per 50 *μ*L squirt. Each dose consists of two squirts, one to each nostril [23]. Most patients started with 1 or 2 doses per hour, which may be increased up to the maximum of 40 doses per day. Nasal irritation is a known disadvantage of this method of NRT.

## 10. Sublingual Tablet

A nicotine tablet has been developed and is marketed in many European countries but not yet in the United States. The tablet is to be held under the tongue, where the nicotine in the tablet is absorbed sublingually. The levels of nicotine obtained are comparable to the gum [24]. It is recommended that smokers use the product for at least 12 weeks, after that the number of tablets used is gradually tapered.

## 11. New Developments to Increase the Efficacy of NRT

### 11.1. Combination of NR Products

Often, a smoker willing to quit does not get the adequate replacement by a single form of NRT. One of the commonly used methods to address this issue is to combine different forms of NRT products, more commonly, combining gum with a patch [12]. Evidence from a meta-analysis shows that combining a nicotine patch with an oral form of NR was more effective than a single type of NRT [25].

However, not only the dose received by the patients that is important to reduce the craving and acute withdrawal symptoms but also the rapidity of rise in blood nicotine concentration following the dosing is vital. It has been found that the nicotine nasal spray, though it delivers less nicotine than nicotine gum, reduces craving faster than 4 mg gums due to its fastest uptake [26].

Recently, some new formulations from NicoNovum (Helsingborg, Sweden) have been tested: a mouth spray and a small teabag-like pouch are to be fitted in under the upper lip against the gum. In studies on smokers abstinent for 1 day, lozenge, mouth spray, and pouch were compared with the Nicorette gum 4 mg on craving, withdrawal symptoms, and preference variables. The study population tried all of the products (mouth spray, lozenge and pouch) with a wash-out period in between. It was found that 2 mg nicotine from mouth spray (1 mg per actuation) and 4 mg nicotine from the pouch reduced craving more and faster than the 4 mg gum and were significantly more liked than the 4 mg Nicorette gum [12].

### 11.2. When and How to Initiate NRT?

Though there is no consensus, the patients are instructed to use one gum every 1 to 2 hours for the first 6 weeks and then to reduce use to one piece every 2 to 4 hours for 3 weeks and one piece every 4 to 8 hours for 3 weeks. Smokers are also advised to use extra pieces between doses in response to episodes of acute craving. Smokers who use less than 25 cigarettes per day are instructed to use the 2 mg dose, and those who smoke more are instructed to use the 4 mg dose. In highly dependent smokers, the 4 mg is superior to the 2 mg gum [11].

Usually patients are instructed to choose a quit date and from that day onwards they are asked to use only NRT and no other tobacco products. In case of any acute craving, they are instructed to take additional dosage as required. Up to relatively recently, the labelling of NR products has mandated that they could only be used after cessation. Again, studies also explored the possibility of starting NRT before the quit day. Four randomized control trials have tested precessation familiarization with NRT. Many of these studies showed improved long-term cessation rates for precessation use [27–29]. A pragmatic randomized control trial on 1100 adult smokers revealed that using NRT 2 weeks before the target quit day was safe and well tolerated but offered no benefit over usual care. However, in conjunction with previous precessation trials there appears to be a moderate benefit, but not as large as that seen in most smaller trials [29].

A recent meta-analysis evaluated the incremental efficacy of starting nicotine patch treatment prior to cessation, compared with the current regimen of starting patch treatment on the target cessation day. It was documented that precessation patch treatment produced a significant increase in cessation rates at 6 months compared to current regimens starting patch treatment on the day of cessation [30].

### 11.3. How Efficacious Are NRTs?

Evidence for NRT effectiveness comes from more than 100 placebo-controlled trials with final follow-up 6–12 months after the start of treatment. Meta-analyses of these trials give odds ratios supporting active treatment ranging from 1.7 to 2.3 according to NRT product [31]. In one review, it was found that NRT improves cessation rates at one year by approximately 70% [7]. In another review, it was shown that outcomes after only 6–12 months of follow-up, as used in existing reviews and treatment guidelines, will overestimate the lifetime benefit and cost-efficacy of NRT by about 30% [32]. Because the long-term benefit of NRT is modest, tobacco dependence treatment might be better viewed as a chronic disorder, requiring repeated episodes of treatment [32].

## 12. Bupropion

It was originally marketed as an antidepressant medication. But subsequently it was also found to be helpful in managing other conditions. Bupropion is chemically unrelated to tricyclic antidepressants or selective serotonin reuptake inhibitors (SSRIs). The mechanism of action as an antidepressant is poorly understood; presumably it involves dual inhibition of dopamine and norepinephrine reuptake in both the mesolimbic dopaminergic system and the locus ceruleus of the brain [33]. It has been hypothesized that the increased levels of dopamine and norepinephrine in these areas simulate the reward achieved when tobacco is used and reduce withdrawal symptoms when tobacco use is stopped [34]. Again the mechanism of action in smoking cessation may be related to its effects on dopamine reward pathways or to inhibition of nicotinic acetylcholine receptors [35]. It is also possible that bupropion acts by reducing some of the symptoms of nicotine withdrawal, which includes depression [11]. The USA Clinical Practice Guideline recommends Bupropion as a first line therapy for nicotine cessation [4].

Various studies have documented the role and efficacy of bupropion in smoking cessation. Over a period of 2 weeks, 300 mg of bupropion significantly reduced abstinence-associated increase in rated depression, difficulty in concentrating and irritability and attenuated a decrease in positive effect, relative to placebo [34]. The researchers also found that the medication might have a positive effect on performance measures during the withdrawal period. However, they did not notice any effects on craving, anxiety, restlessness, or hunger. It has also been found that bupropion combined with nicotine replacement medications may increase rate of abstinence relative to bupropion alone [36]. The efficacy of bupropion is found to be related to the dose used, mean plasma drug concentration, and the blood concentration of the drug metabolites [37]. Smokers who used bupropion at a dose of 100 mg, 150 mg, or 300 mg daily were 1.42, 1.69, and 2.84 times more likely to quit smoking, respectively, than those who used placebo [37]. Clinical trials have also shown that the bupropion is equally efficacious in both men and women [33]. A recent study has found that those smokers who metabolised nicotine faster had relatively better outcome. This study suggests that slow metabolisers had equivalent cessation rates with placebo or bupropion (32%) and fast metabolisers had low cessation rates with placebo (10%) but significantly higher rates with bupropion (34%) at the end of the 10-week treatment phase. However, at the 6-month follow-up, the relationship between the speed of metabolism and cessation remained similar, but differences were no longer statistically significant [38]. In some earlier clinical trials, a modest effect of bupropion SR on reducing weight gain during the drug treatment phase was observed, but no sustained effect was appreciated [33].

Three formulations of bupropion are available: immediate release (taken three times daily), sustained release (taken twice daily), and extended release (taken once daily). Mean half-life of bupropion is about 12 hours, ranging from 8 to 40 hours. The 24-hour exposure occurring after administration of the extended-release version of 300 mg once daily is equivalent to that provided by sustained release of 150 mg twice daily. Clinically, this permits the drug to be taken once a day in the morning. The recommended and maximum dose of bupropion is 300 mg/day, given as 150 mg twice daily [11]. Dry mouth and insomnia are the common adverse events associated with bupropion use. Insomnia occurs in 30% to 45% of bupropion SR users at a dosage of 300 mg/d. This adverse effect is found to be related to dose used and is more common at higher doses [33]. The other minor side effects are anxiety, nausea, and headache. There is also an increased chance of seizures with the use of bupropion, the incidence ranging from 0.1 to 0.4%. The seizure risk with bupropion is higher for the immediate-release form of the drug when it is given at doses of 450 mg or more [33]. The usual length of treatment is 6–12 weeks, but bupropion can be used safely for much longer [39].

## 13. Varenicline

Varenicline is an *α*_4_ *β*_2_ nicotinic receptor partial agonist for smoking cessation. Varenicline was developed to have a high affinity for *α*_4_ *β*_2_ nAChR in the mesolimbic dopamine system and to act as a selective partial agonist of the *α*_4_ *β*_2_ nAChR. Binding at *α*_4_ *β*_2_ nAChR is considered to decrease the craving for nicotine and to relieve the symptoms of withdrawal (agonist effects). Additionally, blocking of nicotine's binding at these receptors is hypothesized to reduce nicotine-induced dopamine release and, consequently, its rewarding/reinforcing effects (antagonist effects) [40].

Varenicline has already received US Food and Drug Administration (FDA) approval for smoking cessation [41]. The recommended use of varenicline has been 0.5 mg daily for 3 days, 0.5 mg b.i.d. for 4 days, and then 1 mg b.i.d. for 11 weeks, and cessation is to occur during week two [42]. The half-life of varenicline is 24 hours. Maximal plasma concentration is achieved within 3-4 hours after administration, and a steady-state concentration is reached within 4 days. The oral bioavailability of this medication is not affected by food or time of administration. It can be administered once daily. But in the clinical setting, varenicline treatment can be optimized by reducing doses in patients who experience intolerable side effects, increasing the dose in partial responders and providing long-term maintenance therapy for relapse prevention [43].

Varenicline has higher abstinence rates than placebo and the alternative active treatments at the end of standard regimen treatment periods. Significantly higher abstinence rates were also found with varenicline in comparison to both placebo and bupropion SR at the end of a 40-week nontreatment follow-up period. Varenicline typically tripled the abstinence rates compared with placebo. In addition, varenicline reduced craving and withdrawal symptoms as well as some of the positive experiences associated with smoking to a greater extent than placebo, bupropion SR, and nicotine replacement therapy (NRT) [44].

However, despite initial encouraging results, the interpretation of these studies should be judged by limitations. Many of these studies were sponsored by pharmaceutical companies. The adverse effect profile of varenicline included nearly 30% of participants reporting nausea, significantly higher proportion of patients reporting abnormal dreams [41]. Moreover, varenicline is suspected to exacerbate depressedmood, as well as erratic and possible suicidal behavior. In 2008, the FDA issued a warning linking varenicline to serious neuropsychiatric symptoms emphasizing the need for alternate therapy, such as immunopharmacotherapy, to aid smoking cessation [8]. A meta-analysis of 14 double-blind randomized controlled trials involving 8216 participants has shown that varenicline was associated with a significantly increased risk of serious adverse cardiovascular events compared with placebo (1.06% in varenicline group versus 0.82% in placebo group) [45]. Still, varenicline appears to be a promising medication in management of nicotine dependence.

## 14. Other Medications for Treating Nicotine Dependence

### 14.1. Nortriptyline

Although the U.S. Food and Drug Administration (FDA) has not approved nortriptyline for use in smoking cessation, the Tobacco Use and Dependence Clinical Practice Guideline Panel of the U.S. Public Health Service recommends it as a second-choice medicine for this use [4]. Six placebo controlled trials have shown that nortriptyline doubles the quit rate as compared to placebo and the efficacy did not appear to be related to its antidepressant action [46].

### 14.2. Clonidine

*Clonidine* is an *α* noradrenergic agonist that suppresses sympathetic activity and has been used for hypertension and to reduce withdrawal symptoms associated with misuse of alcohol and opiates. Both in its oral and low dose patch formulation, clonidine increased smoking cessation in eight out of nine trials, but the drug is associated with serious side effects, including sedation and postural hypotension. Clonidine is therefore probably best reserved for smokers who cannot or do not wish to use NRT, bupropion, or nortriptyline [47].

### 14.3. Nicotine Vaccines

Efforts have been made to produce antibodies against the nicotine molecule that prevents the drug from reaching neural receptors that produce the effects normally associated with smoking.

A significant number of nicotine haptens have been reported. Additionally, various types of delivery vehicles have been used which range from traditional carrier proteins such as KLH, recombinant cholera toxin B subunit, and pseudomonas exoprotein A to a 19-residue conformationally biased peptide that eliminates the need for external adjuvant. Finally, virus-like particles derived from Qb bacteriophage have been used [9].

The vaccine stimulates the immune system to produce antibodies against nicotine, and the nicotine-antibody molecules are too large to pass from the blood into the brain [48]. Theoretically, by eliminating the amount of nicotine reaching brain, one would reduce the reinforcing property of tobacco smoking, eventually leading to extinction of the behavior. However, since the amount of nicotine reaching brain is reduced rather than completely eliminated, there are possibilities that some smokers would actually increase tobacco consumption, at least in the short term, to achieve the levels of nicotine normally obtained during smoking. Results also suggest that a nicotine vaccine would be useful as a relapse prevention treatment [11].

Preclinical studies of short- and long-term administration of nicotine found that one of the nicotine vaccines reduced the distribution of nicotine into the brain in rats by up to 65% [48]. There are at least three companies in early clinical development of an antinicotine vaccine: Xenova (TA-NIC), Nabi (NicVAX), and Cytos (Nicotine-Qbeta) [47]. Active immunization was done with 2 to 6 doses in a period of 2 to 4 weeks plus a later boost for NicVAX and TA-NIC. The serum antibody levels increased after each subsequent dose and were maintained over a couple of months, however, only via subsequent booster dosage. Phase I results for all three vaccines revealed that the formulations were safe and well tolerated with only mild local and systemic reactions that subsided without medical intervention. However, results from large scale phase II trials have been released for NicVAX and NicQb showing limited efficacy obtained to date [9]. In another randomized controlled trial, the most prevalent local adverse event was pain at the injection site and the most frequent systemic adverse event was transient flu-like symptoms which were self-limiting [49].

It has been found in a clinical study that was designed to test safety in an escalating dose design demonstrated that more subjects with high antibody responses quit smoking during the trial than those with lower antibody responses [50]. It is also worth mentioning here that several authors have noted that vaccines might be used as a prevention technique in youths that do not smoke or are experimenting [51].

### 14.4. Rimonabant

Rimonabant, a selective type 1 cannabinoid receptor (CB1) antagonist, may assist smoking cessation by restoring the balance of the endocannabinoid system, which can be disrupted by prolonged use of nicotine [52]. During treatment, overweight or obese smokers tended to lose weight, while normal weight smokers did not. Early in 2006, the FDA issued a nonapprovable letter for the smoking cessation indication; thus, further studies may be required before the FDA will reconsider approval of rimonabant for smoking cessation [52]. Several countries have placed legal restrictions on the compound.

## 15. Conclusion

Nicotine dependence syndrome is a burning problem of the present world considering its impact on health and morbidity. Several medications have been tried including age old nicotine replacement therapies, bupropion, and varenicline. Recent endeavor in this regard is a nicotine vaccine which is yet to gain approval for routine application.
